# Supplementary material for: Effects of light spectra and 15N pulses on growth, leaf morphology, physiology, and internal nitrogen cycling in Quercus variabilis Blume seedlings
Source: PLoS One. 2021 Jul 15;16(7):e0243954. doi: 10.1371/journal.pone.0243954 (PMC8282041; doi:10.1371/journal.pone.0243954)
Supplement: Attachment — Submitted filename: 2021.3.15 Rebattal letter(1).docx [file pone.0243954.s002.docx]

Responses to Review Comments:

Please use the space provided to explain your answers to the questions above. You may also include additional comments for the author, including concerns about dual publication, research ethics, or publication ethics. (Please upload your review as an attachment if it exceeds 20,000 characters)

Reviewer #1: The manuscript has a novel title and rich indicators, which has a certain guiding significance for nitrogen efficient utilization of Quercus variables. However, there are some problems in writing：

75-77 The results of the above-mentioned studies demonstrate a knowledge gap that can be easily filled by new observations with studies using LED and N pulse to mimic natural conditions. What new observations?

[Response] New observation refers to studies concerning the interaction between light spectra and N deposition in the understory condition. This term is not necessary and needs to be removed.

86-90 What are the hypotheses based on before the experiment? It seems not mentioned in the introduction.

[Response] Generally the hypotheses should be put forward on the basis of current findings. It is naturally to expect to see the link between hypotheses to synthesized studies. However, the topic in this study that lighting spectra impact on inherent nutrient retranslocation has no basis that can be referred to. Please see the end of the paragraph that summarized the LED lighting on plant nutrition, it has been indicated that “… further explanation of the percent and amount of inner-derived nutrients by exposure to LED spectra is lacking” on Line 67 and 68.

92 What is the test time (year, month)?

[Response] The study was commenced at early May and ended up in mid-October. All time courses have been added.

136 It can be seen from Figure 1 that the seedling growth (number of leaves, plant height) under the three visible light colors are not consistent, whether it will affect the test results? In the process of the experiment, should we choose the seedlings with the same growth status as far as possible?
[Response] Yes, it is natural that hard wood seedlings have significant individual differences. This is due to the genetic difference from ontogeny for seedling variation. The episodic growth pattern will also contribute to the difference among individuals. This influence can be even more effective on physiology than genotype. All these natures have been well detected and established by the study group on hardwood regeneration in Purdue University. Please refer to one of their successional studies, Sloan and Jacobs (2016). The individual difference will generate some impact on results, not only in our study, but also for all studies if seedlings were taken as the materials. However, this type of impact belongs to the systematic error that can be avoided or reduced by statistics, replicates and means comparison. This is why some results fail to follow the expectation and show no difference. We cannot choose seedlings with uniform size or we will ruin the rule of statistics of random sampling. The screening for the even size has been done on the stage of transplant. Anyway, we have found some significant results that can support our hypotheses. This means that our statistics are fine to cope with systematic errors. However, we add something about this error to discussion.

Reference:

Sloan, JL; Jacobs, DF. Ontogeny influences developmental physiology of post-transplant *Quercus rubra* seedlings more than genotype. Annals of Forest Science, 73(4): 987-993.

218 There are many indicators in the result, but the writing is confusing. It is suggested to clarify the levels of the indexes from three aspects: 1. Leaf morphology and physiological indexes; 2. Plant growth and biomass distribution; 3. Plant nitrogen content and distribution. Meanwhile, it is suggested to use appropriate statistical methods to explain the relationship between indicators (such as correlation analysis).

[Response] Thanks the parameters have been re-classified again according to the suggested orders and sequences. We added a last section of ‘Derived-Nitrogen from Internal and External Sources’. We wished to use Pearson correlation to explain inter-relationship between variables at the initial stage of this article composition. However, we found we cannot achieve this analysis. This is because the core and most valuable meaning of this study was that we revealed internal and external N sources in oak seedlings subjected to different N and Light effects. Leaf, growth, biomass, and generally total N content variables were calculated for both controlled and N-deposited seedlings. However, derived N sources were calculated as the difference between deposition and control. This means that it is important to match N-source data to any other variables.

138 What is the basis for such distribution from table 1？

[Response] As we explained in lines 123 to 126. These spectra were setting according to aims to enable ordinary growth for tree plants with uniform light photons, intensity, flux density, but just different spectra. We have listed eight citations that together support the basis of data in Table 1.

267 What is the significance of analyzing N concentration and N content separately? If there is no specific significance, it is suggested to delete one section of the analysis.

[Response] N concentration, as it is shown in Figure 5, is the percentage of N amount on the unit weight of biomass. N content, shown in Figure 6, is the product of N concentration and biomass. Therefore, technically they are two different things and derived different sources of variables due to the involvement of biomass product. We cannot remove either of them because N concentration is the precondition to read results about N-derived percentage and N content about N-derived amount.

250 It is suggested to combine table 2 and table 3 at the beginning of result.

[Response] These two tables have been combined together.

290-316 Poor writing. Since there is no difference in the experiment, there is no need to spend a lot of time on it. The obvious experiment is meaningless. It is suggested that the discussion should be shortened. Moreover, the experimental data seems to be only one year, without repeated results for many years, which seems to be too hasty.

[Response] The first two paragraphs have been largely shortened. We did conduct a specific experiment with all environmental conditions controlled in the laboratory. We did not have to repeat our experiment in another as it is the necessary task for field studies. The necessity of cross-year experiment is needed for field investigation that would be largely impacted by unexpected episodes, such as extreme weather, unforeseen pest or bacterial infection, anthropogenic damage, and natural fires. Our study employed the classical methodology for a fully controlled experiment to detect the specific response of internal nitrogen cycling although sometimes nothing has been changed from outside. At least 20 studies have been published and obtained excellent citation records including those we refer to, such as Warren et al. [1], Salifu et al. [2], Wei et al. [3], Li et al. [4], He et al. [5].

References:

[1] Warren CR, Livingston NJ, Turpin DH. Response of Douglas-fir seedlings to a brief pulse of ^15^N-labeled nutrients. Tree Physiol. 2003; 23: 1193-1200.

[2] Salifu KF, Islam MA, Jacobs DF. Retranslocation, plant, and soil recovery of nitrogen-15 applied to bareroot black walnut seedlings. Commun Soil Sci Plant Anal. 2009; 40: 1408-1417.

[3] Wei HX, Xu CY, Ma LY, Wang WJ, Duan J, Jiang LN. Short-term nitrogen (N)-retranslocation within *Larix olgensis* seedlings is driven to increase by N-deposition: Evidence from a simulated N-15 experiment in Northeast China. Int J Agric Biol. 2014; 16: 1031-1040.

[4] Li XW, Chen QX, Lei HQ, Wang JW, Yang S, Wei HX. Nutrient uptake and utilization by fragrant rosewood (*Dalbergia odorifera*) seedlings cultured with oligosaccharide addition under different lighting spectra. Forests. 2018; 9: 15.

[5] He CX, Zhao Y, Zhang JS, Gao J. Chitosan oligosaccharide addition to Buddhist Pine (*Podocarpus macrophyllus* (Thunb) Sweet) under drought: Reponses in ecophysiology and delta C-13 abundance. Forests. 2020; 11: 13.

317-381 For the difference of seedling growth caused by different light color, the depth of discussion is not enough, and no more novel content is obtained. I think this is the place that needs to be improved. These discussions are not enough to explain the hypothetical problems. Previous studies should be cited from multiple perspectives (physiological, molecular) to explain the differences.

[Response] We have revised the discussion part. The novelty of our study is that we are the first to reveal derived-N sources in response to the combination of N deposition and light spectra.

Reviewer #2: The authors studied the effects of light spectra (blue, red, and green spectra) and 15N pulses on Quercus variabilis Blume seeds. The idea is clear and the work is relative rich, but it is still far from publication and needs a major revision:

1. Please polish the language carefully.

[Response] After the revision of this round we will sent our manuscript to some professional agency to modify the language.

2. The title of this manuscript may need to be changed, because you have also spent a lot of space describing the effects of light spectra and 15N pulses on other indicators of plants, such as Growth and Leaf Morphology, Leaf Physiology, Biomass Accumulation and Allocation.

[Response] We modified our title according to this suggestion.

3. The contents of Table 2 and Table 3 were basically expressed in the following figures. Do not express the same contents both in figures and tables, please delete or modify.

[Response] I am sorry but data in Table 2 and 3 are P-values from ANOVA and according figures are results of difference which was preconditioned and ruled by Tables 2 and 3. However, we combined the two tables.

4. line 220-223: Here, these treatments have no significant effect on plant indexes, but the changes of indexes are mentioned later. Please rewrite to make it clear.

[Response] This is an error. Amended.

5. line 224: Maybe it should be "higher" instead of "lower"?

[Response] Yes it should be. Amended. Thanks.

6. line 260: The "most" here is not rigorous, there are only two seedling tissue parts except for acorn. Please describe clearly.

[Response] Thanks for correcting. Amended.

7. line 276-279: This paragraph is obscure, please rewrite it.

[Response] It has been rewritten.

8. line 282-284 and line 286-288: The sum of the percent of NDFP and the percent of NDFR is 100%, so if one is high, the other must be low. There is no need to repeat this known fact.

[Response] Thanks. The latter part has been removed.

9. The Discussion section of this manuscript is very poorly written, please take the time to revise the discussion section carefully. The discussion section is used to discuss the important results of the study and their significance. I really don’t understand why the authors spent several paragraphs to write about the effects that they failed to find. You listed a bunch of insignificant examples (such as line 293-296) and made many unimportant literature comparisons, however, you did not discuss the important findings of this study in depth. This made the topic of this paper and the main scientific questions of the study were not clear.

[Response] The first two paragraphs have been largely shortened to avoid over too much discussion about unchanged results. We transferred the main attention in discussion to findings as the important part.

10. line 323-325: please rewrite this sentense.

[Response] Rewritten.

11. line 330: Should it be "not significantly" instead of "significantly"?

[Response] Thanks. This sentence has been revised.

12. line 346-347: You said "another study" but didn't cite the literature.

[Response] Citation added.

13. line 356: Missing citation after "other studies".

[Response] Other studies have been removed.

Responses to Katalin Lorincz:
PLOS ONE

Thank you for submitting your manuscript entitled "Effects of light spectra and 15N pulses on growth, leaf morphology, physiology, and internal nitrogen cycling in Quercus variabilis Blume seedlings" to PLOS ONE. Your manuscript files have been checked in-house but before we can proceed we need you to address the following issues:

I.) It appears that your ORCiD iD has not been validated in your Editorial Manager account and we are unable to proceed until that step is complete.

To validate your ORCiD iD in Editorial Manager, please follow the steps below:

1. In your Editorial Manager account, please go to ‘Update my Information’ (in the upper left-hand corner of the main menu), and click on the Fetch/Validate link next to the ORCiD field.

2. This link will take you to the ORCiD site and allow you to create a new iD or authenticate a pre-existing iD in Editorial Manager.

For additional instructions, please watch the following video for a step-by-step demonstration: https://www.youtube.com/watch?v=_xcclfuvtxQ
[Response] ORCiD has been validated in our Editorial Manager system.

II.) To comply with PLOS ONE submissions requirements for field studies, please provide the following information in the Methods section of the manuscript and in the “Ethics Statement” field of the submission form (via “Edit Submission”):

a) Provide the name of the authority who issued the permission for each location (for example, the authority responsible for a national park or other protected area of land or sea, the relevant regulatory body concerned with protection of wildlife, etc.). If the study was carried out on private land, please confirm that the owner of the land gave permission to conduct the study on this site.

[Response] This statement has been incorporated into the M&M part: Authors stated that the name of the authority who issued the permission for field acorn collection was Nanshan Forest Farm, Jiyuan City, Henan Province, China.

b) For any locations/activities for which specific permission was not required, please

[Response] Not the case.

- i. state clearly that no specific permissions were required for these locations/activities, and provide details on why this is the case

[Response] Not the case.

- ii. confirm that the field studies did not involve endangered or protected species

[Response] Not the case.

c) For vertebrate studies only, please provide the following additional information:

[Response] Not the case.

- i. Full details of collection and sampling methods, including method of sacrifice if applicable

[Response] Not the case.

- ii. State whether the vertebrate work was approved by an Institutional Animal Care and Use Committee (IACUC) or equivalent animal ethics committee. If no approval was obtained, please explain why it was not required.

[Response] Not the case.

- iii. State clearly whether all sampling procedures and/or experimental manipulations were reviewed or specifically approved as part of obtaining the field permit.

[Response] Not the case.
