# Supplementary material for: Effects of light spectra and 15N pulses on growth, leaf morphology, physiology, and internal nitrogen cycling in Quercus variabilis Blume seedlings
Source: PLoS One. 2021 Jul 15;16(7):e0243954. doi: 10.1371/journal.pone.0243954 (PMC8282041; doi:10.1371/journal.pone.0243954)
Supplement: Attachment — Submitted filename: 2021.5.24 Rebattal letter.docx [file pone.0243954.s003.docx]

PONE-D-20-37547R2
Effects of light spectra and 15N pulses on growth, leaf morphology, physiology, and internal nitrogen cycling in Quercus variabilis Blume seedlings
Dr. Chunxia He

Dear Dr. He,

Thank you for submitting your manuscript entitled "Effects of light spectra and 15N pulses on growth, leaf morphology, physiology, and internal nitrogen cycling in Quercus variabilis Blume seedlings" to PLOS ONE.  Your manuscript files have been checked in-house but before we can proceed we need you to address the following issues:

Thank you for updating your data availability statement. You note that your data are available within the Supporting Information files, but no such files have been included with your submission. At this time we ask that you please upload your minimal data set as a Supporting Information file, or to a public repository such as Figshare or Dryad.

[Response] We have uploaded our raw data to the submission system as Supporting Information files.

Please also ensure that when you upload your file you include separate captions for your supplementary files at the end of your manuscript.

[Response] We also mention our supplement of raw data in supporting information at the end of our manuscript.

As soon as you confirm the location of the data underlying your findings, we will be able to proceed with the review of your submission.


Your manuscript has been returned to your account. Please log on to PLOS Editorial Manager at https://www.editorialmanager.com/pone/ to access your manuscript.

Your manuscript can be found in the "Revisions Sent Back to the Author" link under the New Submissions menu. After you have made the changes requested above, please be sure to view and approve the revised PDF after rebuilding the PDF to complete the resubmission process. 

Please note that these changes have been requested to comply with submission guidelines and your manuscript will *not* be sent to review until you have fully adhered to our requests.  Once your paper has been seen by an Editor we may return it to you for further information or amendments.

We ask that you address this request within 21 days. If you require additional time, please email the journal office. We are happy to grant extensions of up to one month past this due date. If we have not heard from you within 21 days, your manuscript will be withdrawn from Editorial Manager. 

Thank you for submitting your work to PLOS ONE.

Kind regards,

Orsolya Voros
PLOS ONE

PONE-D-20-37547R1
Effects of light spectra and 15N pulses on growth, leaf morphology, physiology, and internal nitrogen cycling in Quercus variabilis Blume seedlings
PLOS ONE

Dear Dr. He,

Thank you for submitting your manuscript to PLOS ONE. After careful consideration, we feel that it has merit but does not fully meet PLOS ONE’s publication criteria as it currently stands. Therefore, we invite you to submit a revised version of the manuscript that addresses the points raised during the review process.

[Response] Thanks for giving the revision decision. We will process of manuscript with suggested changes accordingly.

==============================
Dear authors, based on the revisions provided by three referees, I should inform that your manuscript cannot be accepted for publication in this current form and “major revisions” is needed.

Although reviewer 1 think that all comments have been fully addresses, reviewer 2 argued that the authors did not fully address his/her comments. As can be seen below, reviewer 2 pointed out the necessity to reorganize the introduction (to get the three hypotheses) and discussion. Reviewer 2 also complained about the language usage.

[Response] We will specially pay attention to concerns and marks of reviewer 2 and revise the manuscript to the best extent to meet his/her requirements.

I kindly invite you to provide a revised version of your manuscript including the suggestions and comments of all reviewers, particularly the comments of reviewer 2. Together with your resubmission, please provide a point by point account of your revisions specifying how and where you addressed each suggestion. It is important not only to give answers in your response letter, but also to make the appropriate changes in the manuscript, wherever they are appropriate. Thus, my final decision will be taken after your revised manuscript has been reviewed by reviewers again.

[Response] We will revise the manuscript according to comments and suggestions.

In the previous response letter, the authors replied that “After the revision of this round we will sent our manuscript to some professional agency to modify the language”. I would strongly suggest that the authors polish the language before they submit the revised manuscript.

[Response] Thanks for remaindering. We will polish the language before revising the manuscript.

Best regards,

Xiao Guo

Academic Editor

==============================

Please submit your revised manuscript by Jun 19 2021 11:59PM. If you will need more time than this to complete your revisions, please reply to this message or contact the journal office at plosone@plos.org. When you're ready to submit your revision, log on to https://www.editorialmanager.com/pone/ and select the 'Submissions Needing Revision' folder to locate your manuscript file.

Please include the following items when submitting your revised manuscript:

- A rebuttal letter that responds to each point raised by the academic editor and reviewer(s). You should upload this letter as a separate file labeled 'Response to Reviewers'.
- A marked-up copy of your manuscript that highlights changes made to the original version. You should upload this as a separate file labeled 'Revised Manuscript with Track Changes'.
- An unmarked version of your revised paper without tracked changes. You should upload this as a separate file labeled 'Manuscript'.

If you would like to make changes to your financial disclosure, please include your updated statement in your cover letter. Guidelines for resubmitting your figure files are available below the reviewer comments at the end of this letter.

If applicable, we recommend that you deposit your laboratory protocols in protocols.io to enhance the reproducibility of your results. Protocols.io assigns your protocol its own identifier (DOI) so that it can be cited independently in the future. For instructions see: <http://journals.plos.org/plosone/s/submission-guidelines#loc-laboratory-protocols>. Additionally, PLOS ONE offers an option for publishing peer-reviewed Lab Protocol articles, which describe protocols hosted on protocols.io. Read more information on sharing protocols at <https://plos.org/protocols?utm_medium=editorial-email&utm_source=authorletters&utm_campaign=protocols>.

We look forward to receiving your revised manuscript.

Kind regards,

Xiao Guo, Ph.D.
Academic Editor
PLOS ONE


[Note: HTML markup is below. Please do not edit.]

Reviewers' comments:

Reviewer's Responses to Questions

**Comments to the Author**

Please use the space provided to explain your answers to the questions above. You may also include additional comments for the author, including concerns about dual publication, research ethics, or publication ethics. (Please upload your review as an attachment if it exceeds 20,000 characters)

Reviewer #1: (No Response)

Reviewer #2: The manuscript is still poorly written: the language is still irregular, and the statement is still unacademic.

[Response] We will polish our English language before revisions were made and promoted again after revision.

The significance of this study needs to be summarized again. If the significance of this study only showed that red-light spectrum should be avoided for Q. variabilis regenerations, this study is too limited to be publish in this journal.

[Response] Significance has been reconsidered and revised with a new expression in Abstract and Introduction parts. Current significance in scientific contribution reads limited because it concerns the results on species in Abstract. Therefore, Abstract will be revised to reveal results about deep mechanism for scientific contributions. Introduction and Discussion will be revised accordingly. The scientific contribution of this study was to test the modification of internal vs outside sources of derived N that was conducted on Q. variabilis seedlings.

How did you get the hypotheses of this study? The three hypotheses were gotten without any reasoning.

[Response] Hypotheses have all been replaced by new ones that are supported by cited studies.

The discussion section has not been improved. It is still not highlighting the significance of this study, no focus, no main line, logical confusion.

[Response] The whole discussion part has been totally revised.

Reviewer #3: In this study, the authors examined the effects of different light spectra and 14 N pulses on morphology, physiology, and nitrogen (N) uptake performance of Quercus variabilis Blume seedlings. I think this manuscript was generally well designed and analyzed. But still there are some incorrect and awkward sentences that need to be double checked.

[Response] Thanks for indicating flaws of our manuscript. We will revise the sentences and double checked them accordingly with the revision by marks of other reviewers and editors.

L54 Which classic model of the relationship?

[Response] The classic model is the exponential fertilization model that was formally put forward by Timmer in 1997. Here one explanation was lacked to indicate that, in this model, dilution status of nutrients in seedlings was caused by accelerated biomass accumulation without fertilizer supply. The biomass accumulation can be promoted by a prolonged photoperiod, but fertilizer regime was not changed, which together resulted in the dilution. We revised this part.

L65 Is the effect of this mixed spectrum consistent with the single spectrum?

[Response] In the range of cited studies, we can say yes.

L71 Maybe the research background can be put in the first paragraph

[Response] Yes it should be. Changes made with proper modifications.

L89, 90 What are the experimental hypotheses based on? It's not completely consistent with the results of others research (line 62-66). Maybe you can change it to questions.

[Response] No, it meets results of studies in 62-66. Dilution induced by blue light results from promoted biomass accumulation without enough nutrient supply, but that by green light was the result of insufficient nutrient supply. However, we know that we did not reveal enough background to link current hypotheses to core meanings of background. We revised the hypotheses to meet the meaning on paper.

L149 Only one tanked pot of seedlings received 15N pulsing? One in four? What is the number of total replications of seedlings received 15N pulsing?

[Response] Only the four potted seedlings in one tank of a growing floor received 15N pulse leaving the other four receiving just water. This part is unclear. So, it is revised. Totally, 108 seedlings received pulse and the other 108 ones received water. Either pulsed or controlled seedlings were arranged in 3 replicates as three shelves for one spectrum.

L210-210 Paragraph spacing is different from before

[Response] Changes made.

L231 Please hang the first line

[Response] Changes made.

L270 Describe results that are significantly different firstly, rather than no significant

[Response] Changes made.

L310 But there was a significant influence of light on aboveground biomass in table 2

[Response] acorns grew underground which was not affected by the aboveground biomass.

L 276-297: I suggest modifying these phrases. First describe what you found and then the possible explanation with citations, rather than a lot of citations. You can check that along all discussion.

[Response] Number of citations will be reduced with additions of more explanations that will help further diluting the density of citations. This will be made throughout the discussion.

Table 2 I suggest marking the P-values less than 0.05 with asterisks or letters

[Response] Thanks and asterisks will be used.

7. PLOS authors have the option to publish the peer review history of their article ([what does this mean?](https://journals.plos.org/plosone/s/editorial-and-peer-review-process#loc-peer-review-history)). If published, this will include your full peer review and any attached files.


If you choose “no”, your identity will remain anonymous but your review may still be made public.


**Do you want your identity to be public for this peer review?** For information about this choice, including consent withdrawal, please see our [Privacy Policy](https://www.plos.org/privacy-policy).

Reviewer #1: No

Reviewer #2: No

Reviewer #3: No

[NOTE: If reviewer comments were submitted as an attachment file, they will be attached to this email and accessible via the submission site. Please log into your account, locate the manuscript record, and check for the action link "View Attachments". If this link does not appear, there are no attachment files.]

While revising your submission, please upload your figure files to the Preflight Analysis and Conversion Engine (PACE) digital diagnostic tool, <https://pacev2.apexcovantage.com/>. PACE helps ensure that figures meet PLOS requirements. To use PACE, you must first register as a user. Registration is free. Then, login and navigate to the UPLOAD tab, where you will find detailed instructions on how to use the tool. If you encounter any issues or have any questions when using PACE, please email PLOS at [figures@plos.org](mailto:figures@plos.org). Please note that Supporting Information files do not need this step.

*In compliance with data protection regulations, you may request that we remove your personal registration details at any time.* [*(Remove my information/details)*](https://www.editorialmanager.com/pone/login.asp?a=r)*. Please contact the publication office if you have any questions.*
